# Heat Processing Reduces IgE Binding but Not Basophil Sensitivity to Pea Proteins in Pea-Allergic Children

**DOI:** 10.3390/nu18101612

**Published:** 2026-05-19

**Authors:** Malgorzata Teodorowicz, Anja E. M. Janssen, Joyce Emons, Willemijn Lissenberg, Anouk Verstappen, Janneke Ruinemans-Koerts

**Affiliations:** 1 Department Cell Biology and Immunology, Wageningen University and Research Centre, 6700 AH Wageningen, The Netherlands; willemijnlissenberg@gmail.com (W.L.); anouk.verstappen@hotmail.com (A.V.); janneke.ruinemans-koerts@wur.nl (J.R.-K.); 2 Food Process Engineering Group, Wageningen University and Research Centre, 6700 AH Wageningen, The Netherlands; 3 Department of Paediatrics, Division of Respiratory Medicine and Allergology, Erasmus University Medical Centre—Sophia Children’s Hospital, 3015 GD Rotterdam, The Netherlands; 4 Department of Clinical Chemistry, Haematology and Immunology, DICOON BV, location Rijnstate Hospital, 6815 AD Arnhem, The Netherlands

**Keywords:** pea allergy, peanut allergy, food processing, Western blot, IgE profile, IgE binding, indirect basophil activation test (iBAT), Pis s 1, PA2 a/b

## Abstract

Background/Objectives: The increasing use of pea protein in plant-based foods raises concerns about IgE-mediated reactions, particularly in individuals sensitized to peanut. Knowledge on clinically relevant pea allergens and the impact of heat processing remains limited. This study investigated how thermal treatment affects the IgE binding and functional allergenicity of pea proteins in children with a confirmed pea allergy, with or without a concomitant peanut allergy. Methods: Serum from 11 patients was analyzed using SDS-PAGE, Western blotting, and an indirect basophil activation test (iBAT). Results: All patients showed IgE binding to Pis s 1 and PA2a/b in raw pea extract, with variable sensitization to Pis s 2 and mitogenic lectin. Heating (120 °C, 5 min) markedly reduced IgE binding and eliminated detectable IgE to Legumin S and ML. Despite this reduction, basophil sensitivity did not decrease; in several patients, EC50 values significantly decreased, indicating increased basophil responsiveness to heated pea. Patients with IgE profiles dominated by Pis s 1 and PA2a/b were most likely to show enhanced basophil activation after heating. Conclusions: These findings demonstrate that heat-stable vicilin subunits and albumins can maintain functional allergenicity despite reduced IgE recognition, underscoring the need for diagnostic approaches that incorporate processed food allergens.

## 1. Introduction

The demand for sustainable protein sources is growing, as animal protein consumption is decreasing due to its significant environmental impact, particularly its contribution to greenhouse gas emissions and land use, as well as potential health risks like increased saturated fat and cholesterol intake. The most commonly used plant-based protein sources that substitute animal protein are legumes (like peanut, pea, soy, beans and lentils), wheat (seitan), tree nuts and seeds [1,2]. Pea is a preferred protein source due to its ability to closely mimic the structure of meat proteins under appropriate processing conditions, while its flavor and natural diversity in size, shape, and color further enhance its suitability for applications in the meat replacement industry [3]. Despite the nutritional advantages of plant-based proteins, there are concerns about the risk of IgE-mediated allergic reactions. Increased consumption of concentrated (novel) plant proteins may lead to a rise in sensitization rates and, consequently, to more allergic reactions in both individuals with no previous allergic complaints and patients with sensitizations/allergies to cross-reactive allergens [4]. In particular, the high number of people with a peanut allergy might be at risk, as there is a potential for IgE cross-reactivity between allergens from different legume species which can be clinically relevant. Remarkably, pea allergenicity has mainly been described in children with peanut sensitization [5,6,7,8,9,10]. To define patients who are at risk of pea allergy, more knowledge is required about the major pea allergens involved and, in particular, whether the same pea allergens/epitopes are involved in patients with an additional peanut allergy [11]. Secondly, current in vitro diagnostic tests are based on raw pea extracts, while pea is mostly eaten after thermal processing. Food processing, like heating, might change its allergenicity, as has been observed in a few pea-allergic patients by a skin-prick test or oral exposure [12,13]. While processing can modify existing epitopes, it can also generate neo-epitopes with the potential to trigger food allergies [14]. Consequently, evaluating the impact of processing on the inherent allergenicity of food proteins remains essential. It is crucial to recognize that processing-induced alterations in the degree of IgE antibody binding may not necessarily translate into changes in a protein’s overall allergenicity or its capacity to induce sensitization [15]. However, which pea allergens/epitopes are susceptible to heat processing and for whom this might have clinical consequences remain unknown. By introducing novel sustainable plant-based proteins, reliable in vitro diagnostics will enhance the consumption of plant-based foods, preventing unnecessary diet restrictions because of fear of severe allergic reactions.

The major pea allergens described so far are Pis s 1, a vicilin of 44 kDa; Pis s 2, a convicilin of 63 kDa; and Pis s 3, an nsLTP of 9.5 kDa. Pis s 1 and Pis s 2, also regarded as storage proteins, can be proteolytically degraded into different subunits (α, β and y), which can also be allergenic [9]. Co-sensitization between different legumes is high but infrequently accompanied by clinical symptoms. The main allergens responsible for these co-sensitizations are Pis s1 with peanut Ara h 1 and Pis s 3 with the nsLTP from peanut (Ara h 9) [16]. Much less is known about the potential allergenicity of the pea albumin fraction, i.e., PA1 and PA2, and their subunits, a and b, due to proteolysis. Cross-reactivity of these pea albumins within the legume family is unknown. Other proteins which are known for sIgE binding are Legumin S and Mitogen Lectin (ML), although their clinical relevance is also largely unknown [17].

Given the high prevalence of peanut allergy and the increased risk of clinical reactivity to pea in peanut-allergic individuals compared with non-peanut-allergic subjects, there is a clear need to better define which patients are at risk by identifying the specific (cross-reactive) allergens involved [5,6,18]. Additionally, knowing that pea proteins are predominantly consumed in processed forms and that food processing may modify allergenicity, understanding the effects of processing on the allergenicity of pea proteins is highly relevant [12,13].

So far, research on the clinically relevant (cross-reactive) pea allergens has mainly been established by detection of allergen sIgE binding or inhibition profiles, either via immunoblot or (inhibition) IgE-ELISA. However, allergen sIgE levels cannot predict clinical symptoms; therefore, an oral exposure test (food challenge test) is considered as the gold standard, but in research settings this test is frequently hampered due to ethical restrictions. In this situation, an in vitro functional basophil assay like the basophil activation test (BAT) is a good and practical alternative [19,20]. The aim of this study is to determine, in pea-allergic patients with or without a peanut allergy, the major pea allergens in raw and heated pea extract and, secondly, whether heating, as a form of food processing, can influence the allergenicity of pea. To our knowledge, this is the first paper in which possible changes in the allergenicity of pea protein due to thermal processing has been established by comparison of the allergen sIgE profile (Western blot) with an in vitro functional indirect basophil activation test (iBAT) using serum from pea-allergic patients with and without a peanut allergy.

## 2. Materials and Methods

### 2.1. Patient Serum and Clinical Data

Sera of children with a pea allergy were selected from the biobank of the Children’s Allergy department of the Erasmus Medical Centre. Clinical and laboratory data were collected (see Table 1). Pea allergy diagnosis was established with pea sensitization, in addition to an allergic reaction in the patient’s history or a positive food challenge test. Patients were selected and categorized into 2 groups: 1. pea allergy with peanut sensitization (n = 6) and 2. both pea and peanut allergy (n = 7). Specific IgE (sIgE) levels for pea and peanut (and its specific allergen components) were measured using either the ImmunoCAP250 (Thermo Fisher Scientific, Waltham, MA, USA) and ALEX2^®^ (Allergy Xplorer, Micro Array Diagnostics GmbH, Vienna, Austria) in kU/L (>0.35 kU/L is regarded as positive test), or the ImmunoCAPTM ISACTM (Thermo Fisher Scientific, Waltham, MA, USA) in ISAC standardized units for IgE (ISU-E) (>0.3 is regarded as a positive test). SPT was measured in Histamine Equivalent Prick results with a threshold for a positive test of >0.4. All sera were recruited, stored and analyzed according to the protocol approved by the Medical Ethical Committee of Rijnstate Hospital and the Erasmus Medical Centre (nos. 2022-2032).

### 2.2. Preparation of Pea Extract and Heat Treatment

Pea protein samples were prepared based on the method of Rivera del Rio et al. [21]. Dry yellow peas (*Pisum sativum* L.; Alimex, The Netherlands) were milled into flour and suspended in Milli-Q water (1:5, *w*/*v*) under stirring for 60 min at room temperature (RT). The suspension was centrifuged (1 s, 1500× *g*, 20 °C) to separate the protein-rich fraction (PRF) from the starch-containing pellet. The PRF was further centrifuged (30 min, 10,000× *g*, 20 °C) to obtain the soluble protein fraction (SPF). The SPF was divided into different treatments: untreated or heated at 120 °C for 5 min (after reaching the target temperature). After heating, samples were centrifuged (20 min, 10,000× *g*, 10 °C) and supernatants were collected and stored at −80 °C for further analysis.

### 2.3. SDS-PAGE

Protein concentrations were equalized across samples prior to SDS–PAGE analysis, and 30 µg of each pea protein extract was loaded into 4–15% Mini-PROTEAN^®^ TGX precast gels (Bio-Rad Laboratories, Hercules, CA, USA, #4561083). Precision Plus Protein™ Dual Color Standard (Bio-Rad, #1610374EDU) was included as a molecular weight indicator. Prior to loading, the samples were mixed with Laemmli sample buffer containing β-mercaptoethanol and heated at 90 °C for 5 min to denature proteins and reduce disulfide bonds. Electrophoresis was performed in Tris–glycine–SDS running buffer (25 mM Tris, 192 mM glycine, 0.1% SDS). Gels were stained with Coomassie Brilliant Blue, destained with deionized water until background staining was minimal, and imaged using a Bio-Rad Universal Hood III system. Digital image data from electrophoresis gels were visualized, and the molecular weights of the bands were analyzed using Image Lab v. 6.1 software (Bio-Rad).

### 2.4. Western Blot

Western blotting was performed following SDS–PAGE as described above, up to completion of gel electrophoresis. Proteins were transferred onto nitrocellulose membranes (0.45 µm; 0.45 µm; Amersham™ Protran, Cytiva, Marlborough, MA, USA, #GE10600002) using a semi-dry transfer system (Trans-Blot^®^ SD Cell, Bio-Rad). Prior to transfer, gels and membranes were equilibrated for 15 min in semi-dry transfer buffer (25 mM Tris, 192 mM glycine, 0.04% SDS, 20% methanol). Transfer was carried out at 15 V for 35 min. Following transfer, membranes were washed twice with Tris-buffered saline containing 0.05% Tween-20 (TBS-T) and blocked for 1 h at room temperature in 3% bovine serum albumin (BSA) in TBS. Membranes were then washed and incubated overnight at 4 °C with human serum (1:30 dilution) diluted in TBS containing 0.5% BSA under gentle agitation to allow the specific IgE to bind to the allergens. After incubation, membranes were washed four times with TBS-T supplemented with 0.01% Triton X-100. Secondary antibody incubation was performed for 1 h at room temperature using horseradish peroxidase-conjugated goat anti-human IgE (Thermo Fisher Scientific; #A18793) diluted 1:850 in 3% BSA in TBS. Membranes were subsequently washed with TBS-T and TBS to remove unbound antibody. Protein bands were detected using the WesternBright™ enhanced chemiluminescence (ECL) substrate (Advansta Inc., San Jose, CA, USA, K-12045-D20) according to the manufacturer’s instructions. Chemiluminescent signals were imaged using a Bio-Rad Universal Hood III system, and band detection and analysis were performed using Image Lab software (Bio-Rad). Band identification was based on a combination of (i) molecular weight comparison with published data and (ii) alignment of Western blot membranes with the corresponding SDS PAGE gels. Band intensities were quantified by optical density measurement (Bio-Rad, Image Lab v. 6.1). Based on the distribution of OD values in our dataset, bands were classified as weak (<3.0 × 10^5^), medium (3.0 × 10^5^–2.0 × 10^6^), or strong (>2.0 × 10^6^). These thresholds correspond to the natural clustering of OD values and reflect the visual scoring used during initial band identification. For visualization of IgE binding in the heat map, bands were classified based on their optical density and coded as follows: no detectable band = 0, weak = 1, medium = 2, and strong = 3.

### 2.5. Indirect Basophil Activation Test

The indirect basophil activation test (iBAT) was performed according to previously published protocol [20]. Samples consisting of 4 mL aliquots of fresh EDTA-anticoagulated blood (<24 h old) from six adult non-allergic healthy blood donors with the blood group O were centrifuged for 10 min at 2200 g at room temperature. Buffy coats were collected and combined, then washed with physiological salt and resuspended in a total volume of 2 mL (leucocyte count between 12.5 and 15 × 10^9^/L). The resuspended buffy coat was centrifuged for 5 min at 1000 g and 11 °C, after which 2 mL of cold stripping buffer (0.15 M sodium dihydrogen phosphate monohydrate and 0.005 M potassium chloride, pH 3.55) was added to the buffy coat and the centrifuge protocol was repeated. After the stripping procedure, the buffy coat was washed with Basophil Stimulation Buffer (containing calcium, heparin, and IL-3; Bühlmann, Basel, Switzerland). A 500 μL aliquot of buffy coat was incubated with 130 μL of serum from each tested patient for 16 h at 37 °C. Non-Treated protein (Pea NT) and pea heated at 120 °C (Pea H) were added in the same run, using the same batch of patient-resensitized donor basophils. The lowest pea protein concentration was 78 ng/mL, and the maximum concentration was 7500 ng/mL. Based on the BAT results for three peanut-allergic patients with pea IgE sensitization without a recorded pea allergy, %CD63 > 6 (corrected for negative control) was regarded as a positive BAT. The difference in BAT results between Pea NT and Pea H was based on the EC50 value (the concentration of allergen which induces a response halfway between the baseline and the maximum), which is regarded as a measure of basophil sensitivity. Although the AUC (area under curve) can also be used as a read-out parameter for basophil activation (sensitivity and reactivity), this value was less reliable and applicable in this study as for some patients the dose–response curves did not reach a plateau phase. Dose–response curves were fitted in GraphPad Prism (version 8.0.2; GraphPad Software, San Diego, CA, USA) using a three-parameter logistic curve fit (hill slope 1).

## 3. Results

### 3.1. Clinical and Demographic Data of Patients

Twelve patients were included in this study from the outpatient pediatric allergy department in the Sophia Children’s hospital. These patients were mostly boys (75%), young (2–13 years), and diagnosed with multiple food allergies. In total, 85% of the patients were also diagnosed with eczema, 31% with asthma and 85% with allergic rhinitis as an atopic comorbidity. One patient, a boy of 11 years, was selected from Rijnstate Hospital (patient no. 2). The patients were divided into a pea-allergic group (n = 6) and a peanut- and pea-allergic group (n = 7), as shown in Table 1, with known sensitization values for pea and peanut components. Clinical symptoms of pea and/or peanut allergies are reported in Table 1.

### 3.2. SDS-PAGE Profile of Non-Treated and Heated Pea Proteins

**Figure 1:**
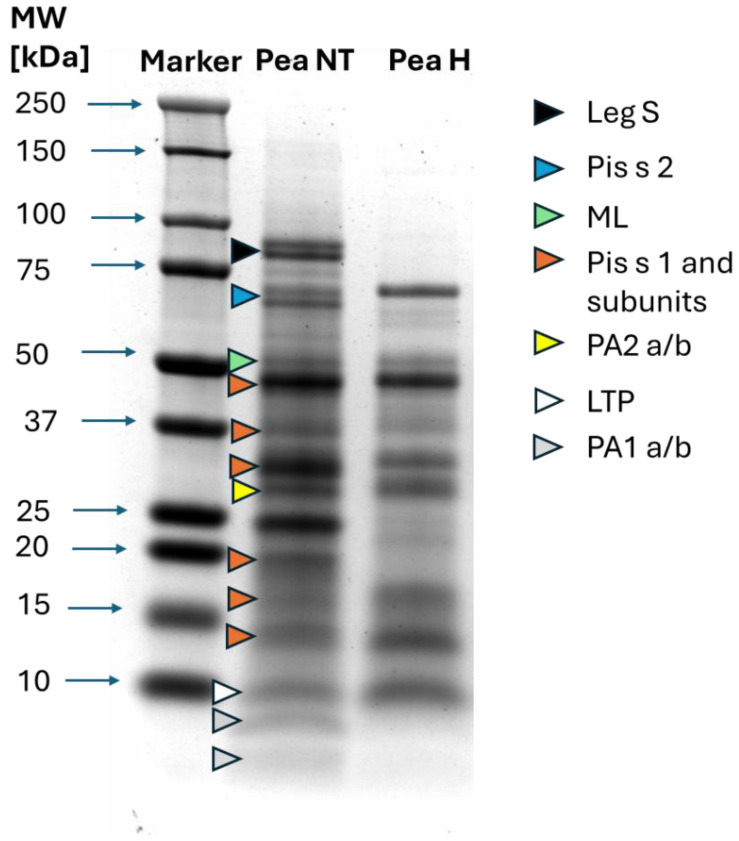
SDS-PAGE picture of non-treated (Pea NT) and heated (Pea H) protein extracts. The colored arrows indicate bands with molecular weights corresponding to the molecular weights of proteins described in the literature. In order, from the top: Legumin (Leg S), Convicilin (Pis s 2), Mitogenic Lectin (ML), Vicilin (Pis s 1) and its subunits (arrows 4–6, 8–10), albumin subunits (PA2 a/b), and LTP and pea albumin (Pa 1 a/b).

The SDS-PAGE analysis revealed the composition of proteins present in the non-treated (Pea NT) and heat-treated (Pea H) pea extracts. Allergen identification was based on the molecular weights reported previously for pea storage proteins and albumins [9,16,22]. In the non-treated extract, five known pea allergens were detected, spanning approximately 80–13 kDa. These included Legumin S, Pis s 2, PA2, mitogenic lectin, and Pis s 1, together with its characteristic subunits. Protein fractions below 10 kDa were visible but remained unidentified because the lower detection limit of the gel system was approximately 10 kDa. All identified bands and their putative allergen assignments are shown in Figure 1.

Heating the soluble protein fraction resulted in pronounced changes in the electrophoretic pattern. High-molecular-weight globulins such as Legumin S and fractions below 10 kDa were strongly reduced or no longer detectable, indicating substantial heat-induced denaturation and loss of solubility. In contrast, several low-molecular-weight, heat-stable fragments—particularly those corresponding to Pis s 1 and albumin-derived proteins—remained visible after heating. Overall, the SDS-PAGE profile demonstrates that thermal processing selectively reduces the abundance of specific pea allergens, particularly legumins and certain albumins, while vicilin-related proteins such as Pis s 1 retain substantial stability. These findings confirm the presence of both heat-labile and heat-stable allergenic fractions within the pea protein extract.

### 3.3. Western Blot Profiles of Pea-Allergic Patients Versus Pea- and Peanut-Allergic Patients

Based on the results of the SDS-PAGE, the protein bands in the Western blot could be assigned to specific pea allergens (see Table 2(A,B)). The sIgE binding patterns were analyzed separately in the two patient groups—the pea-allergic (Table 2(A)) and pea- and peanut-allergic groups (Table 2(B)). Differences in IgE recognition between the non-treated and heated pea extracts were evaluated by comparing the intensity of the Western blot bands. Band intensity was quantified by optical density, and the resulting OD values were clustered to reflect the visual scoring used during initial band identification. All Western blot membranes are available in the Supplementary Materials (Supplementary Figure S1).

#### 3.3.1. Pea-Allergic Patients

All pea-allergic patients without a peanut allergy showed sIgE to Pis s1 and one or more of its large or small subunits, as well as PA2a/b and Legumin S in raw peanut extract. sIgE sensitization to Pis s 2 and ML was variable, from intense positive bands to any bands being visible. In this subgroup of pea-allergic patients, possibly three different sIgE patterns were observed based on the major differences in the presence or absence of bands. For patients 1 and 2, a combination of intense Pis s 2 and ML bands, sensitization to PA2a/b, and no sensitization to Pis s 1 large subunits was observed. For patients 3 and 4, intense bands for Pis s 1 large subunits and PA2a/b and no sensitisation to ML was observed. For patients 5 and 6, sensitization to Pis s1 small and large subunits, ML and PA2a/b was observed. After heating, in all patients, sIgE binding for most allergens decreased, and sIgE binding to Legumin S and ML disappeared. Pis s 2 sensitization was only observed for patients 1 and 2 after heating. Only patient 3 did not show any sensitization to PA2a/b after heating.

#### 3.3.2. Pea- and Peanut-Allergic Patients

All patients with both a pea and a peanut allergy had sIgE to Pis s 1, both to large and small Pis s 1 subunits, as well as PA2a/b and Legumin S in raw pea extract. SIgE to Pis s2 and ML was variable, from strong positive bands to any bands being visible. In this subgroup of pea-allergic patients, three different sIgE patterns were visible. For patient 7, a combination of intense Pis s 2 and ML and sensitization to PA2a/b was observed. For patients 8 to 10, intense PA2a/b, low Pis s 2 and negative ML was observed. For patient 11, intense PA2a/b and sensitization to both Pis s 2 and ML was observed. As for the pea-allergic group, heating of pea decreased all sensitizations, and sIgE binding to Legumin S, Pis s 2 and ML disappeared in all patients. In this group only, patients 9 and 11 did not show any sensitization to PA2a/b after heating.

The sIgE levels for pea extract did not explain the variation in sIgE profiles. For example, patients 1, 2, 4, 7 and 8 all had high pea sIgE levels but showed different sIgE profiles.

### 3.4. Indirect Basophil Activation Test

All pea-allergic patients showed a positive BAT (>6% CD63% expression) for untreated and heated pea, except for patient 3, who did not show basophil activation to heated pea (see BAT curves, Supplementary Figure S2). Table 3 shows the EC50 change due to heating. For patients 1, 2, 5, 7, and 11, the EC50 change was nonsignificant, and for patients 3, 4, 8, 9 and 10 the EC50 change was significant, i.e., there was a decrease in EC50 values, corresponding to an increase in basophil sensitivity after heating of pea. All BAT curves and EC50 values (CI 95%) are available in the Supplementary Materials (Supplementary Figure S2 and Supplementary Table S1).

### 3.5. Correlation of sIgE Pea Profile and Change in EC50 from the Indirect Basophil Activation Test

Although heating of pea decreased sIgE binding to all potential pea allergens, basophil sensitivity did not decrease except for patient 6.

In both the pea-allergic patients and the patients with both a pea and a peanut allergy, a sIgE sensitization profile for Pis s 1, Pis s 2, PA2a/b and ML showed nonsignificant BAT EC50 changes due to pea heating, while patients with a profile for Pis s 1 and PA2a/b, with or without Pis s 2 (see Table 3), showed a significant decrease in BAT EC50 values. Since heating destroyed any sIgE binding to Legumin S and ML and to Pis s 2 in most patients, basophil activation could mainly be attributed to Pis s 1 subunits and/or PA2a/b in heated pea.

## 4. Discussion

This study investigated how heat processing affects IgE binding and functional allergenicity of pea proteins in children with a confirmed pea allergy. Using Western blotting and an indirect basophil activation test, we compared raw and heat-treated pea extracts and identified the allergens most likely to drive reactivity after processing.

All pea-allergic patients were sensitized to the following allergens in raw pea extract: Pis s 1, PA2a/b and Legumin S. More variable sensitization to Pis s 2 and ML was observed. In all patients, heat treatment of the pea extract resulted in reduced levels of sIgE to these allergens, with complete loss of detectable sIgE to Legumin S and ML. However, despite this decrease in sIgE binding, the allergenicity, as assessed by BAT, showed that the sensitivity (EC50) for pea did not decrease after heating, and in some patients it even increased significantly. This decrease in EC50 values was observed in patients with a sIgE pea profile for the raw pea allergens Pis s 1 and PA2a/b without sensitization to ML. In addition, this type of sIgE profile was observed in patients with and without a peanut allergy. These results are in line with a previous publication showing that heating of pea might increase its allergenicity in some patients [13].

Based on Western blot analyses, IgE sensitization to Pis s 1 and its proteolytic subunits was detected in all patients, showing Pis s 1 to be one of the major pea allergens in the studied patients. Pis s 1, a 7S vicilin with a molecular weight of 44 kDa, has previously been described as a major pea allergen with IgE binding reactivity correlated with a whole pea extract [9,22,23]. It is worth noticing that Pis s 1 has been described as a highly heterogenic fraction resulting in the presence of a number of polypeptide fractions/subunits formed due to the production of vicilin polypeptides from several small gene families encoding different primary sequences, differential proteolytic processing, and differential glycosylation [24,25,26]. In our study we also observed several Pis s 1 subunits on SDS-PAGE under the reducing condition corresponding to the following molecular weights: 36, 32, 20, 16 and 13 kDa. This is consistent with Sánchez-Monge’s findings, in which protein subunits of 36, 32, 16, and 13 kDa were identified and confirmed as pea vicilin subunits by *N*-terminal sequencing [22]. A previous analysis of IgE recognition patterns using individual sera from pea-allergic patients demonstrated that mature vicilin (Pis 1; 44 kDa) and its 32 kDa proteolytic fragment represent the major immunoreactive fraction, with sIgE detected in approximately 50% of the tested patients [26]. This was confirmed by another study demonstrating that IgE binding to the proteolytic subunits of Pis s 1 was fully or partially inhibited by pre-incubation with the recombinant mature Pis s 1 [9]. Both of the abovementioned studies also indicated that the proteolytic subunits of Pis s 1 might be clinically relevant, although this was not confirmed with functional assays, nor was the influence of processing on IgE reactivity determined. In our study, sIgE binding to Pis s 1 and its subunits was detected in all patients, confirming the importance of Pis s 1 epitopes in the sensitization profile of pea-allergic children. Although heating significantly reduced the intensity of the Pis s 1 band, its proteolytic subunits remained detectable in 9 out of 11 patients, suggesting that these epitopes may retain clinical relevance after heat treatment and potentially contribute to basophil activation. However, since we used extracts in our BAT, and no patients were sensitized to a single pea allergen, it is not possible to draw conclusions about which epitopes are most important or which epitopes become more exposed after heating.

A second key allergen observed in the Western blot analysis was PA2a/b. PA2a/b consistently shows strong IgE binding before and (weaker but present in 8 patients) after heating, in both groups of patients, indicating PA2a/b as a second important pea protein fraction with potential clinical relevance. PA2 a/b belongs to the group of 2S albumin allergens, a family of small, disulfide-rich seed proteins characterized by high thermal stability and strong allergenic potency, analogous to peanut Ara h 2 and Ara h 6 [27]. However, a highly conserved cysteine motif, a key feature linked to the strong allergenicity of Ara h 2 and 6, was not found in the structure of pea PA2, nor has cross-reactivity between these two proteins been demonstrated [16]. Our Western blot data suggest that sensitization to PA2a/b may contribute to basophil activation, as PA2a/b was the only allergenic fraction detected after heating in one patient (patient 5). This confirms a study of Malley et al., who, based on skin-prick testing in 10 pea-allergic patients, showed that the pea albumin fraction was responsible for positive reactions. Importantly, the allergenic activity of pea albumins remained fully intact after heating, highlighting their heat stability [12]. In contrast, Popp et al. did not identify PA2a/b as a relevant allergen based on IgE binding to raw pea extract and recombinant protein in a cohort of 14 pea-allergic children [9]. Although the presence of PA2 in our study was not confirmed by structural sequencing, a band of approximately 26 kDa was also observed on the Western blot image of one patient in the study by Popp et al., supporting the presence of PA2 on SDS-PAGE at a position corresponding to that observed in our study [9]. Finally, discrepant findings regarding the relevance of PA2 may reflect differences in protein preparation and extraction and variation among pea cultivars—whose storage proteins are encoded by multigene families and occur as multiple isoforms—as well as differences in patient cohorts and sensitization profiles.

We further compared the profiles of sIgE binding to pea allergens between two groups of patients: pea-allergic individuals and patients allergic to both pea and peanut. This comparison is particularly relevant given that sensitization to other legumes is common among peanut-allergic individuals; for example, a study of 195 peanut-allergic children showed sensitization to at least one additional legume in 63.9% of cases, with 15.4% being allergic to peas (Muller, Luc et al. 2022 [6]). This raises the question of whether sensitization profiles for key pea allergens differ between patients allergic only to pea and those allergic to both pea and peanut. However, analysis of IgE binding patterns in our study population did not reveal clear differences between the two groups. In both groups, we observed heterogeneous sIgE binding profiles for Pis s 1, Pis s 2, PA2a/b, ML, and Leg S. Also, in both groups we observed significant changes in BAT EC50 values after heating for some patients. The IgE binding profiles of these patients can be characterized by strong sIgE binding to Pis s 1 (including its subunits) and PA2a/b, but not to ML. A lack of concordance between sIgE binding level and BAT suggests that factors beyond linear IgE recognition, such as epitope conformation and epitope accessibility, contribute to basophil activation. Under the applied reducing SDS-PAGE conditions, the Western blot primarily detects sIgE binding to linear epitopes, as protein denaturation disrupts conformational structures. In contrast, the BAT reflects functional cross-linking of cell-bound sIgE on basophils by an allergen in its native or near-native form, allowing recognition of both linear and conformational epitopes. This fundamental difference in epitope presentation may explain the observed differences between the sIgE and BAT assays, which have been previously described for soy proteins also [28].

Based on the Western blot results, the relevance of linear epitopes appeared to decrease after heating, whereas basophil activation for heated pea remained the same or even increased in some patients from both groups compared to the non-treated pea extract. This finding suggests that conformational epitopes might be more exposed during heating, contributing substantially to functional IgE cross-linking and highlighting their potential clinical relevance in pea-allergic individuals. Although Popp et. all identified and mapped 11 relevant linear epitopes of Pis s 1, the role of conformational epitopes remains to be verified [9]. Lastly, thermal processing of food allergens may expose or generate novel conformational epitopes as a result of structural rearrangements, including disulfide bond reshuffling, alterations in α-helical and β-sheet content, and the formation of random coil structures, thereby potentially modulating allergenic activity [14,29,30,31]. Vissers et al. reported reduced IgE binding capacity but increased degranulation activity of heated Ara h 1, which was attributed to the formation of Ara h 1 aggregates. The authors speculated that aggregates present extended surfaces containing multiple copies of identical IgE epitopes, thereby potentially enhancing IgE cross-linking efficiency [32]. PA2 albumin represents an example of an allergen undergoing such heat-induced structural changes, as it is a β-sheet- and α-helix-rich dimer that has been shown to unfold and form large aggregates upon heating [33]. Therefore, it cannot be excluded that heat-induced aggregation of PA2 contributes to the enhanced basophil activation observed after heating, even though such aggregates are not detectable by SDS-PAGE under denaturing conditions. However, this remains speculative and should be confirmed in follow-up studies examining different heating conditions (e.g., baking or drying), including matrix effects, since the heating applied in this study was relatively short and performed in the liquid phase.

This study has several limitations that should be considered when interpreting the findings. The number of participants was small, which limits the statistical power and reduces the generalizability of the observed IgE binding patterns and basophil responses. The Western blot analysis relied on semi-quantitative measurements of band optical density, meaning that differences in IgE binding should be interpreted as comparative rather than absolute. In addition, the identification of allergenic proteins was based on their apparent molecular weight, without structural confirmation by mass spectrometry. These methodological constraints indicate that the results should be viewed as exploratory and highlight the need for validation in larger cohorts. Nonetheless, the consistent patterns observed across patients provide meaningful first insights into how heat processing affects pea allergenicity.

## 5. Conclusions

In conclusion, our study showed that heat treatment markedly reduced IgE binding to several major pea allergens, including Legumin S, Pis s 2, and ML. Despite this reduction, basophil sensitivity did not decrease, since several patients showed significantly lower EC50 values after heating, indicating increased basophil responsiveness. This divergence between IgE binding and basophil activation highlights that reduced IgE binding does not necessarily translate into reduced allergenicity. Heat-stable proteins—particularly vicilin subunits (Pis s 1 fragments) and albumins (PA2a/b)—likely remain capable of cross-linking IgE on basophils. Although a change in the IgE binding reported in the Western blot results is not a good predictive factor for allergenicity, it allows mapping and comparison of the sensitization profiles of raw versus heated pea protein extracts. Patients with IgE profiles dominated by Pis s 1 and PA2a/b were most likely to show increased basophil sensitivity after heating. In contrast, patients with broader sensitization patterns (including Pis s 2 and ML) tended to show no significant change in EC50 results. This suggests that the relative contribution of heat-stable allergens may determine the functional outcome after processing. Lastly, as consumption of processed pea proteins increases, understanding which allergens remain active after heating is essential. Our findings suggest that diagnostic tests based solely on raw extracts may underestimate the allergenic potential of processed pea proteins. Incorporating heat-treated extracts or purified heat-stable allergens into diagnostic platforms may improve risk assessment.

## Figures and Tables

**Table 1:** Clinical information of the study population: A—pea-allergic patients (1–6), B—pea- and peanut-allergic patients (7–11). sIgE and ALEX values were expressed in kU/L, ISAC values were expressed in ISU-E, and SPT values were expressed in mm (millimeters), >0.4 indicating a positive diagnosis. Abbreviations used: anaphylaxis (Ax), Allergy Xplorer (ALEX), ImmunoCAP ISAC (ISAC), skin-prick test (SPT).

	Pea-Allergic Group (A)						Pea- and Peanut-Allergic Group (B)				
		P1	P2	P3	P4	P5	P7	P8	P9	P10	P11
Pea	sIgE	35	60.5				33				
	ALEX				37.3	4.5	40.7	31.2	9.0		6.0
	SPT				4.3	2.1		3.4	3.5	1.7	0.4
Peanut	sIgE						>100			>100	0.7
	SPT			2.2				4.4	1.3	6.6	
Ara h 1	ALEX	23.7					46.1		14.0		8.1
	ISAC			1.6	5.9	1.0		9.2		56	
Ara h 2	sIgE		<0.35								
	ALEX	<0.1					39.3		28.8		8.5
	ISAC			<0.3				15		>100	
Ara h 3	ALEX	2.09					33				4.8
	ISAC							6.1		86	
Ara h 6	ALEX	<0.1					30.6		27.1		2.8
	ISAC			0.3				5.9		44	
Ara h 8	ALEX	<0.1					4.0		0.2		<0.1
Ara h 9	ALEX	<0.1					3.7		2.3		<0.1
Symptoms		Angioedema	Swollen eyes and lips	Small amount of pea and direct oral allergy complaints	Angioedema	Urticaria	Never eaten peanut or pea	Peanut: stomach ache, vomiting, itching; Pea: Ax	Peanut: urticaria, vomiting; Pea: urticaria	Peanut: urticaria, laryngeal oedema; Pea: angioedema	Peanut: urticaria, vomiting; Pea: urticaria

**Table 2:** (A,B) A heatmap of IgE binding profiles of pea allergens in non-treated (Pea NT) and heat-treated (Pea H) pea protein extracts and heat-induced changes in allergenicity assessed by Western blotting. IgE reactivity against individual pea allergens, including Legumin (Leg S, 80 kDa), Pis 1 (44 kDa), Pis 1 subunits (α + β/β + γ and α/β/γ chains), Pis 2, convicilin (ML), and PA2a/b, was evaluated in different pea preparations across patient sera based on the measured optical density (OD) of individual bands: A—pea-allergic patients (1–6), B—pea- and peanut-allergic patients (7–11). The OD thresholds for each category were defined based on the distribution of measured OD values across all samples and coded as follows: no detectable band = 0, weak = 1, medium = 2, and strong = 3.

**A. Pea-Allergic Group**													
**Pea Proteins Non-Treated**							**Pea Proteins Heated**						
	**P1**	**P2**	**P3**	**P4**	**P5**	**P6**	**P1**	**P2**	**P3**	**P4**	**P5**	**P6**	**Allergen**
**Allergen**													
Leg S	1	1	1	1	2	2	0	0	0	0	0	0	Leg S
Pis 1	3	3	2	3	2	2	1	0	1	0	0	0	Pis1
Chains (α + β)	0	0	3	3	1	2	0	0	2	1	0	1	Chains (α + β)
Chains (α/β/γ)	2	2	3	0	3	2	2	1	2	0	0	1	Chains (α/β/γ)
Pis 2	3	3	0	2	0	2	1	1	0	0	0	0	Pis2
ML	3	3	0	0	1	1	0	0	0	0	0	0	ML
PA2a/b	2	2	3	3	1	3	2	1	0	1	1	1	PA2a/b

**B. Pea- and Peanut-Allergic Group**													
**Pea Proteins Non-Treated**							**Pea Proteins Heated**						
	**P7**	**P8**	**P9**	**P10**	**P11**		**P7**	**P8**	**P9**	**P10**	**P11**		**Allergen**
**Allergen**													
Leg S	3	1	1	2	2		0	0	0	0	0		Leg S
Pis 1	2	1	2	1	3		0	0	1	1	2		Pis1
Chains (α + β)	1	1	2	3	3		0	0	1	1	2		Chains (α + β)
Chains (α/β/γ)	1	1	1	1	1		1	0	0	1	1		Chains (α/β/γ)
Pis 2	3	1	0	0	2		0	0	0	0	0		Pis2
ML	3	0	0	0	1		0	0	0	0	0		ML
PA2a/b	1	3	3	3	3		1	1	0	1	0		PA2a/b

**Table 3:** Correlation of sIgE pea profiles based on Western blotting and change in EC50 values in the indirect basophil activation test: *a 100-fold difference (2 log-units) with non-overlapping CIs was considered a relevant shift between individual curves*. * Minimal overlapping confidence interval; ** confidence intervals could not be determined. In bold: Fractions with the highest optical density.

Pea-Allergic			Pea- and Peanut-Allergic		
Patient	ΔBAT EC50	sIgE Profile Untreated Pea	Patient	ΔBAT EC50	sIgE Profile Untreated Pea
**1**	Nonsignificant	Pis s 1 small subunit, **Pis s 2, ML**, PA2a/b	**7**	Nonsignificant	Pis s1 small and large subunits, **Pis s 2, ML**, PA2a/b
**2**	Nonsignificant	Pis s 1 small subunit, **Pis s 2, ML**, PA2a/b			
**6**	*nd*	Pis s 1 small and large subunits, Pis s 2, ML, **PA2a/b,**	**11**	Nonsignificant	**Pis s1** small **and large subunits**, Pis s 2, ML, **P2a/b**
**5**	Nonsignificant	**Pis s 1 small** and large subunits, ML, PA2a/b			
**3**	Significant	**Pis s 1 small and large subunits, PA2a/b**	**8**	Significant	**Pis s 1 small and large subunits,** Pis s 2, **PA2a/b**
**4**	Significant	**Pis s 1 large subunit,** Pis s 2, **PA2a/b**	**9**	Significant *	**Pis s 1 small and large subunits, PA2a/b**
			**10**	Significant **	**Pis s 1** small and **large subunits**, **PA2a/b**

## Data Availability

The original contributions presented in this study are included in the article/Supplementary Materials. Further inquiries can be directed to the corresponding author.

## Supplementary Materials

The following supporting information can be downloaded at https://www.mdpi.com/article/10.3390/nu18101612/s1, Figure S1: Western blot membranes obtained using sera from individual patients. Numbered bands indicate the identified allergenic protein fractions. NT—non-treated pea extract; 120—heated pea extract. Figure S2: Results of the indirect basophil activation test (BAT) for each individual patient obtained for non-treated pea extract (Pea NT) and heated pea extract (Pea 120). Table S1: IC50 values obtained in indirect basophil activation test (iBAT) for each individual patients: >>> = >10^14^; ? = could not be determined.

## Abbreviations

ALEX	Allergy Explorer
BSA	Bovine serum albumin
CI	Confidence interval
ECL	Enhanced chemiluminescence
EC50	Half-maximal effective concentration
iBAT	Indirect basophil activation test
ISAC	Immuno Solid-phase Allergen Chip
Leg S	Legumin S
ML	Mitogen Lectin
nsLTP	Non-specific lipid transfer protein
PA	Pea albumin
Pis	Pisum sativum
PRF	Protein rich fraction
RT	Room temperature
sIgE	Specific Immunoglobilin E
SPF	Soluble protein fraction
TBS	Tris-buffered saline
TBS-T	Tris-buffered saline-Tween
