# Supplementary material for: Heat Processing Reduces IgE Binding but Not Basophil Sensitivity to Pea Proteins in Pea-Allergic Children
Source: Nutrients. 2026 May 19;18(10):1612. doi: 10.3390/nu18101612 (PMC13209531; doi:10.3390/nu18101612)
Supplement: Supplementary file 1 [file nutrients-18-01612-s001.zip › nutrients-4213631-supplementary.pdf]

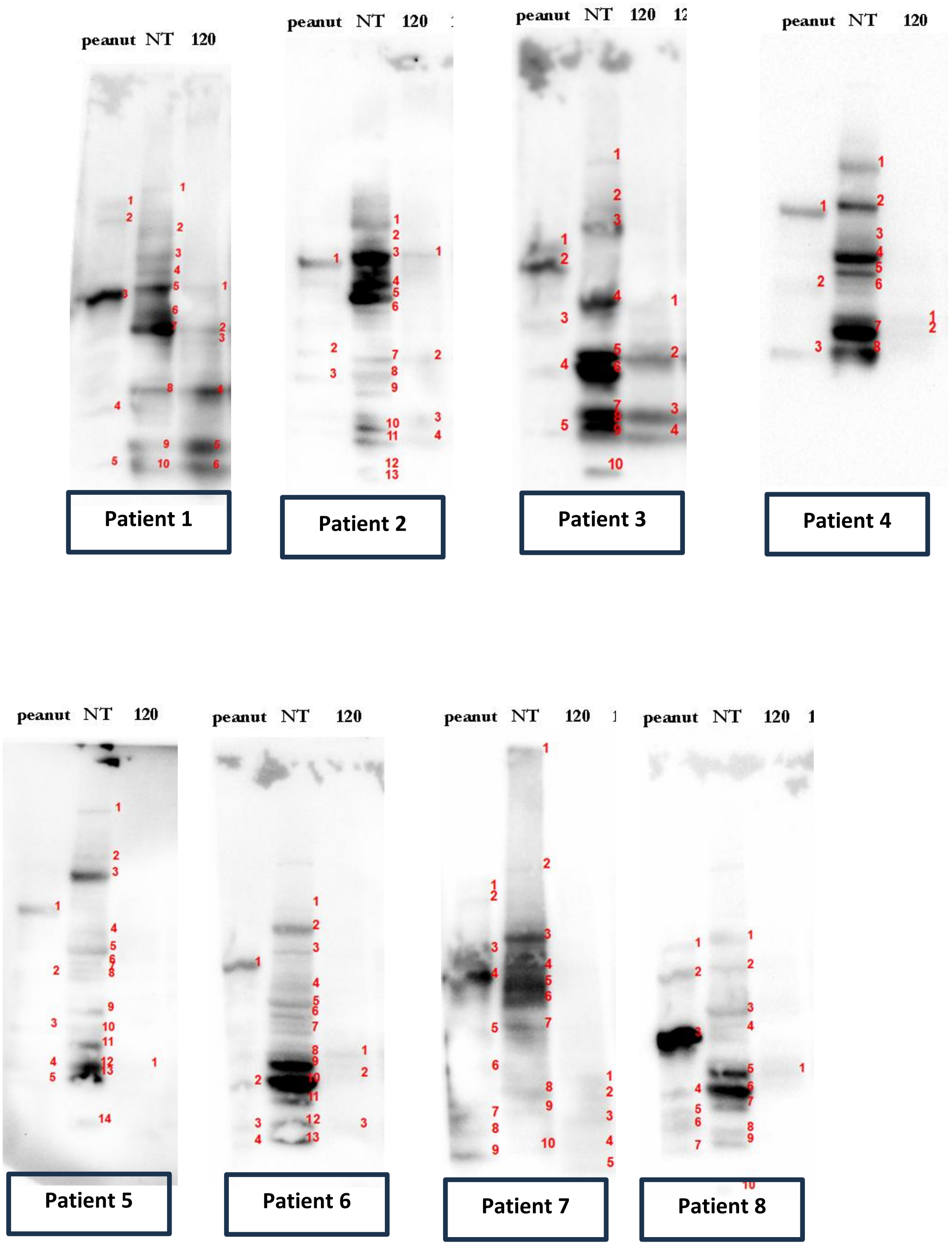

peanut NT 120
Patient 1
peanut NT 120
Patient 2
peanut NT 120
Patient 3
peanut NT 120
Patient 4
peanut NT 120
Patient 5
peanut NT 120
Patient 6
peanut NT 120
Patient 7
peanut NT 120
Patient 8

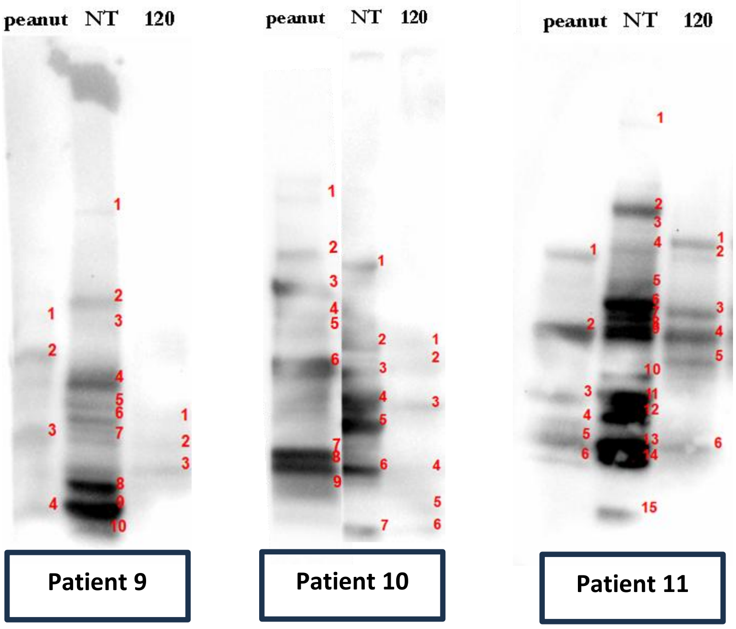


Figure S1: Western blot membranes obtained using sera from individual patients. Numbered bands indicate the identified allergenic protein fractions. NT—non-treated pea extract; 120—heated pea extract.

Patient 4

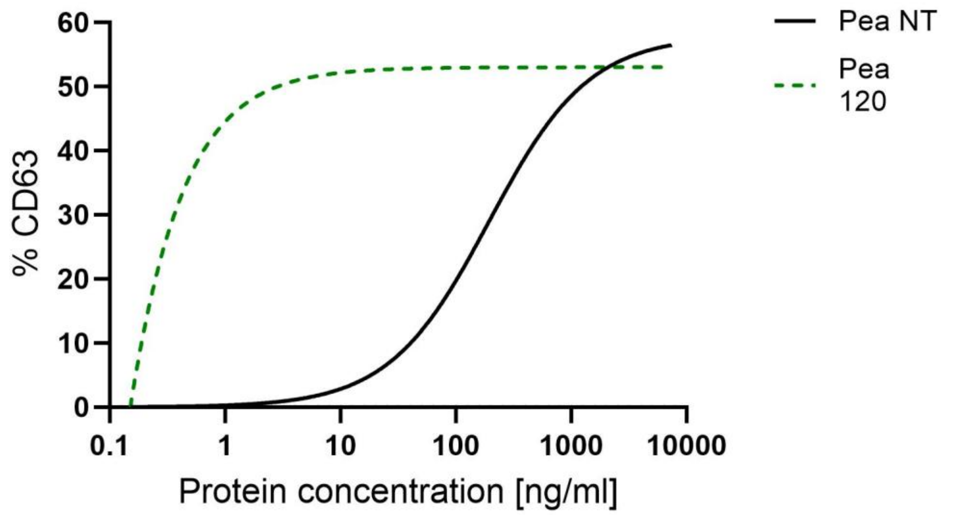


Patient 6

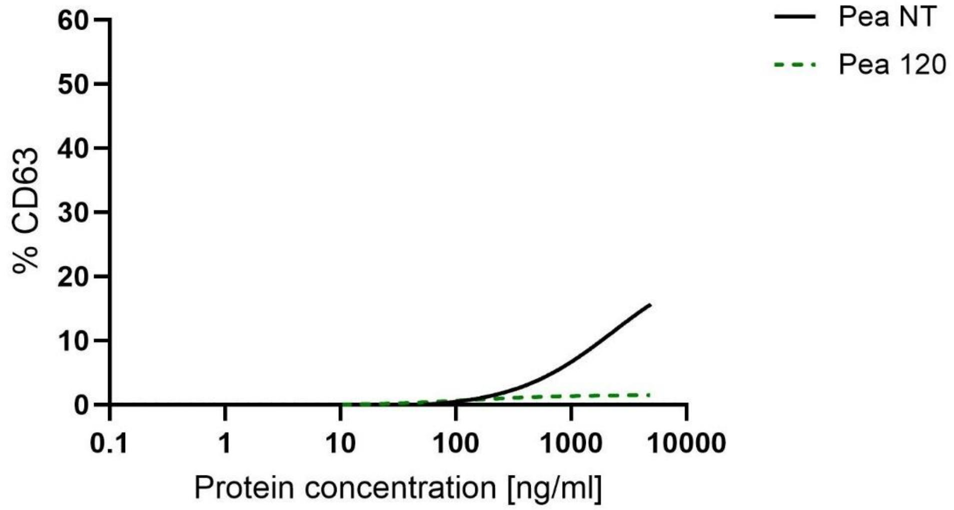

Patient 5

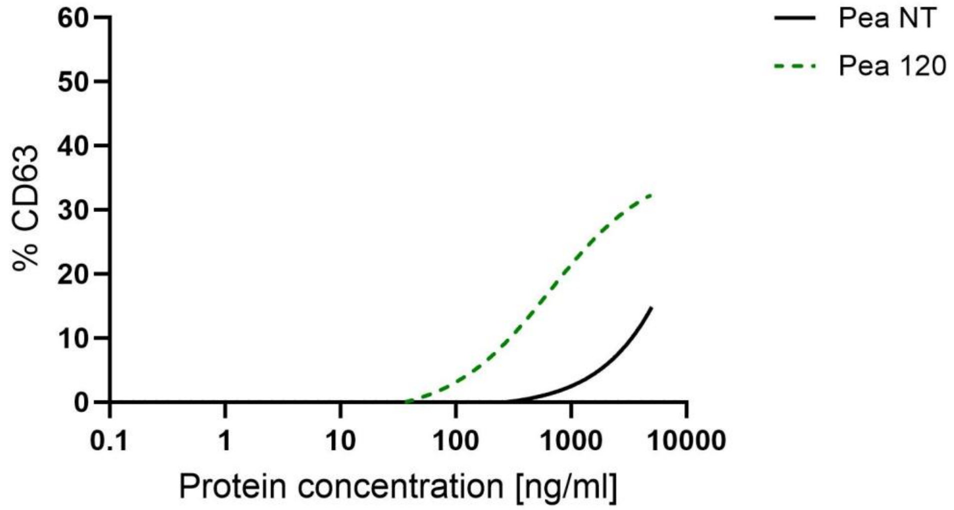


Patient 7

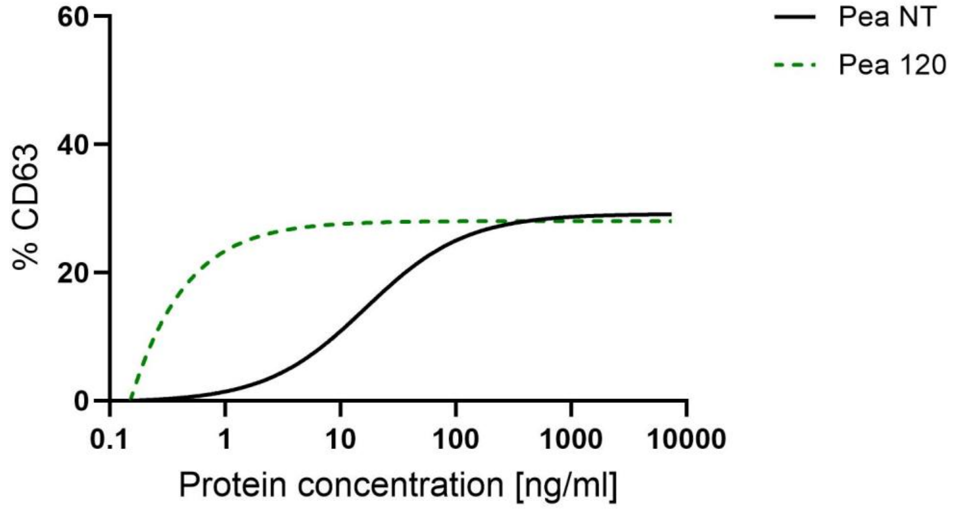

Patient 8

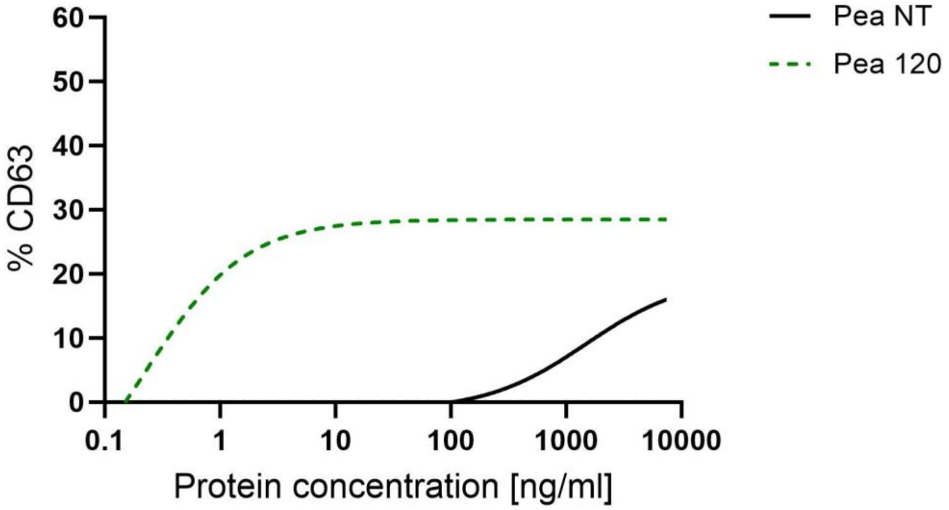


Patient 11

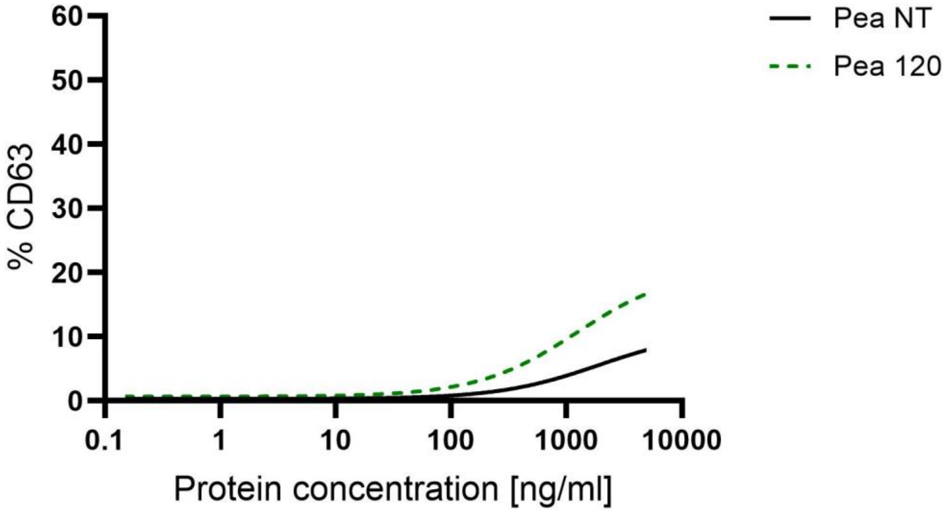

Patient 3

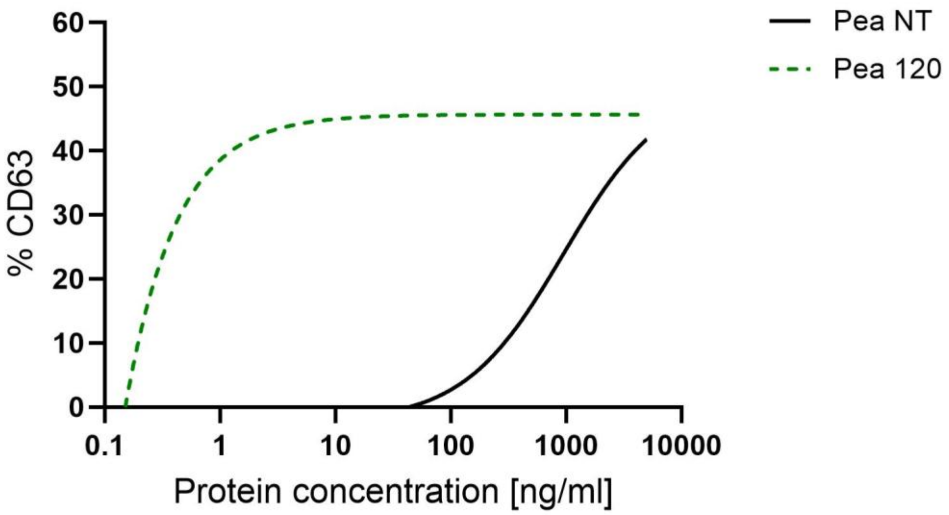


Patient 9

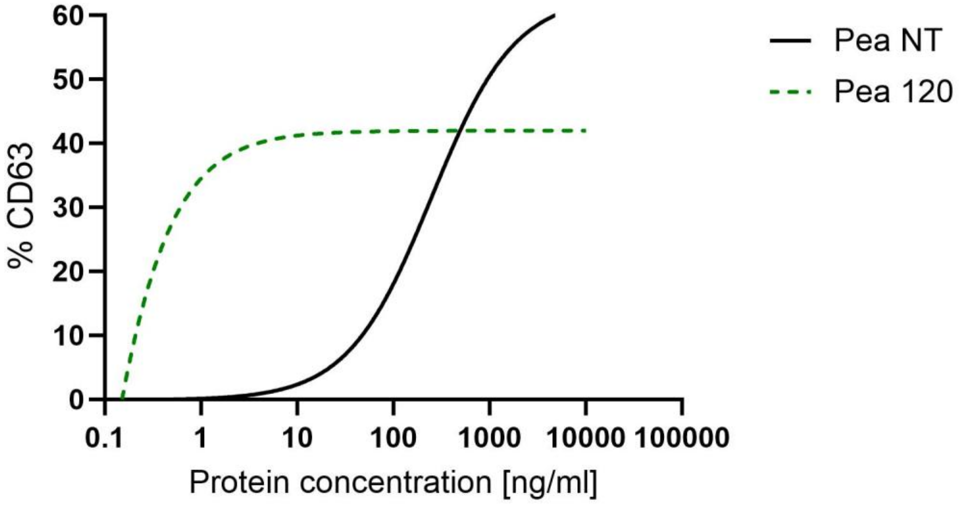

Patient 10

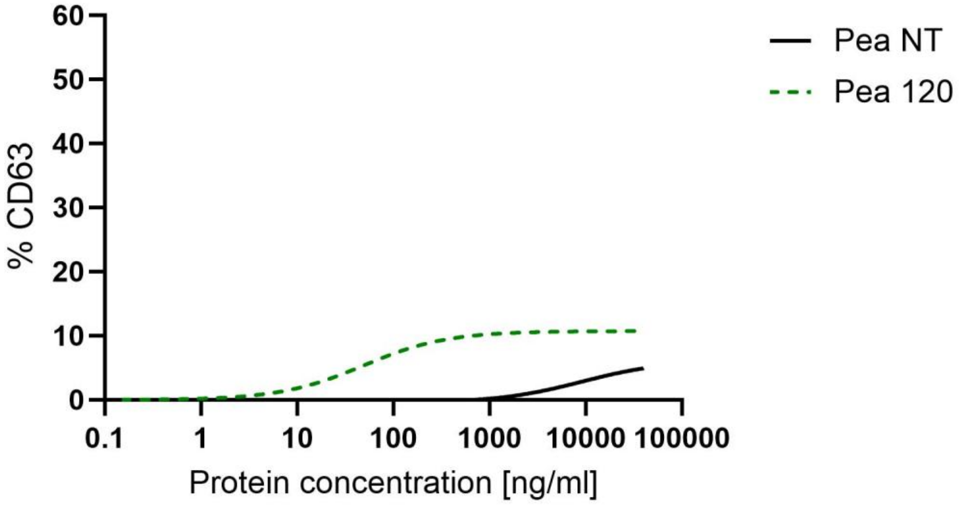


Patient 1

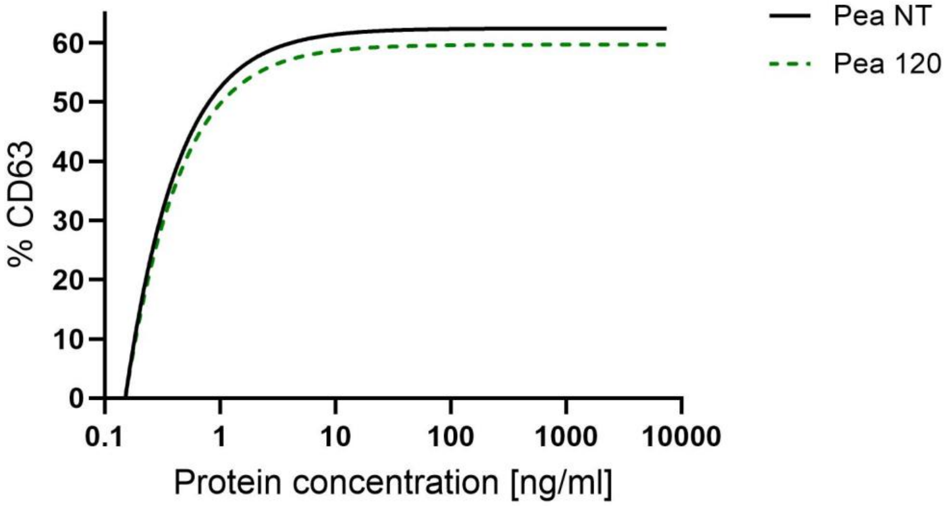

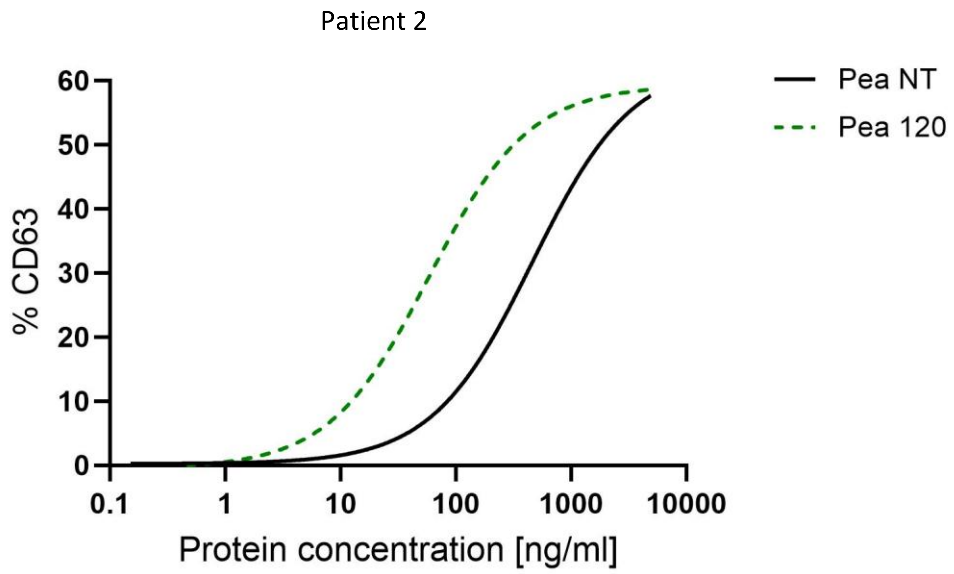


Figure S2: Results of the indirect basophil activation test (BAT) for each individual patient obtained for non-treated pea extract (Pea NT) and heated pea extract (Pea 120).

Table S1. EC50 values obtained in indirect basophil activation test (iBAT) for each individual patients: ? = could not be determined; >>> = >$10^{14}$

| patient | EC50 Pea NT (ng/ml) | CI 95% | EC50 Pea heated (ng/ml) | CI 95% |
|---|---|---|---|---|
| 1 | 775 | ?-35 | 466 | ?-109 |
| 2 | 458 | 378-559 | 58 | 35-90 |
| 3 | 947 | 309-5465 | 0.005 | ?-34 |
| 4 | 194 | 127-280 | 0.012 | ?-58 |
| 5 | 44091 | 788->>> | 658 | 166-5812 |
| 6 | 2309 | 160->>> | 120 | ?->>> |
| 7 | 16 | ?-137 | 0.019 | ?-139 |
| 8 | 1481 | 112->>> | 0.22 | ?-132 |
| 9 | 247 | 103-448 | 0.033 | ?-316 |
| 10 | 9085 | ?->>> | 49 | ?->>> |
| 11 | 1916 | 142->>> | 1237 | 402-7750 |
